# Molar Incisor Hypomineralization (MIH) in a Child with Congenital Chronic Intestinal Pseudoobstruction (CIPO)

**DOI:** 10.1155/2020/8894657

**Published:** 2020-12-30

**Authors:** Mohammed Zameer, Syed Ali Peeran, Syed Nahid Basheer, Syed Wali Peeran, Sameen Badiujjama Birajdar, Faisal Mohammad Alzahrani, Ali Mohammed A. Alkhayrat

**Affiliations:** ^1^Armed Forces Hospital, Jazan, Saudi Arabia; ^2^Department of Restorative Dentistry, College of Dentistry, Jazan, Saudi Arabia; ^3^Department of Periodontology & Oral Implantology, Faculty of Dentistry, University of Sebha, Sebha, Libya; ^4^Sanjeevani Dental Clinic, Raichur, India; ^5^College of Dentistry, Jazan, Saudi Arabia

## Abstract

Molar incisor hypomineralization (MIH) is a qualitative enamel defect of systemic origin affecting 1–4 permanent first molars (PFMs) frequently in association with affected permanent incisors (PIs). The exact etiology of MIH is still unclear but considered to be multifactorial. This present case report to the best of our knowledge is the first case reported which acknowledges MIH in a patient with chronic intestinal pseudoobstruction (CIPO) with underlying neurological disease due to somatic mitochondrial disorder. It also elicits the availability of various contemporary treatment options and their proper selection and early intervention to manage the functional and aesthetic problems caused by enamel defects and to improve the quality of life in the patients.

## 1. Introduction

Molar incisor hypomineralization (MIH) is a qualitative enamel defect of systemic origin affecting 1–4 permanent first molars (PFMs) frequently in association with affected permanent incisors (PIs). Meanwhile, speculation was raised that the tips of permanent cuspids might present similar defects as they mineralized in the same period as the FPMs and PIs [1]. Further in the literature, MIH-like defects have been reported on second primary molars, permanent canines, premolars, and second permanent molars [2–11]. Although the hypomineralization was initially described as MIH, more recently, it has been noticed that these MIH-like defects can affect any primary or permanent tooth [10–13]. MIH defects can be soft, porous, and vary in their clinical presentation from white to yellow or brownish opacities but always with a distinct demarcation between affected and sound enamel. The porous, brittle enamel can cause loss of tooth structure under the masticatory forces [14]. Thus, the enamel porosities and discolored opacities can result in functional and aesthetic complications in MIH-affected children. Other enamel abnormalities like diffuse opacities, hypoplasia, and amelogenesis imperfecta are clinically similar to MIH defects which can also occur during amelogenesis and are suggested to be designated while evaluation to allow distinction from MIH defects [15].

MIH-affected children have also been shown to have an adverse impact on the Oral Health-Related Quality of Life (OHRQoL) along with considerably increased negative self-perception of oral symptoms [16]. For clinicians, the management of MIH cases can be problematic as the affected teeth might be highly sensitive and susceptible to rapid dental caries development. Moreover, limited cooperation of young children, difficulty in achieving anesthesia, and repeated episodes of the marginal breakdown of restorations can complicate their management. Thus, MIH-affected children have shown to spend more time in receiving oral health care in comparison with those without MIH [14, 17, 18].

The exact etiology of MIH is still unclear but is considered to be multifactorial. The causative mechanism of MIH has been associated with both environmental disturbances (prenatal, perinatal, and postnatal disturbances) and genetic factors (genetic variations in TUFT1, TUFT11, AMELX, and ENAM) [19, 20, 21]. To the best of our knowledge, congenital chronic intestinal pseudoobstruction related to underlying neurologic disorder has not been previously described with MIH. The present case report is aimed at narrating the association of congenital chronic intestinal pseudoobstruction (CIPO) with MIH. It also elicits the availability of various contemporary treatment options and their proper selection and early intervention to manage the functional and aesthetic problems caused by enamel defects and to improve the quality of life in the patients.

## 2. Case Report

A 12-year-old boy with his father presented to the Pediatric Dental Clinic of Armed Forces Hospital (Jazan, KSA) with a chief complaint of discoloration in the lower front teeth since their eruption. Medical history revealed that he was born with preterm delivery by cesarean section due to polyhydramnios at the gestational age of 30 weeks. He was hospitalized for 3 weeks due to prematurity and abdominal distension. He was diagnosed to have a chronic intestinal pseudoobstruction, bacterial overgrowth, chronic diarrhea, and metabolic acidosis. He was managed with sodium bicarbonate, potassium chloride, and pancrelipase/Lipase-Protease-Amylase (Creon™), and the bacterial overgrowth was treated with multiple courses of alternating antibiotics: clindamycin, gentamicin, amoxicillin, and metronidazole. At the age of 9 years, the boy again suffered from acute vomiting, diarrhea, and dehydration. The patient was admitted for rehydration, and further investigations were carried out. The patient was diagnosed with pancreatitis and somatic mitochondrial disorder with pyroglutamic aciduria. He also tested positive for rhinovirus. It was inferred that these findings could be pathognomonic to the enteropathy in the patient's underlying neurological disease.

**Figure 1:**
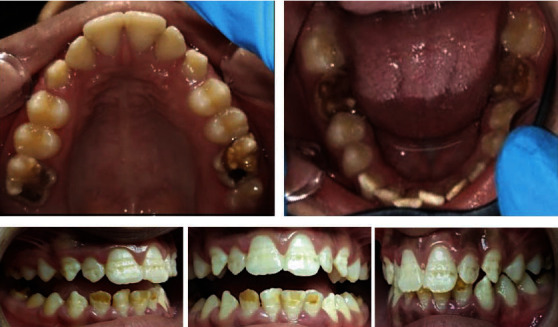
Preoperative intraoral photographs.

Intraoral clinical examination was carried out on clean wet teeth. All four FPMs showed extensive enamel breakdown with irregular brown opacities. Mandibular incisors showed demarcated opacities ranging from white-creamy to yellow-brown defects. These opacities in molars and mandibular incisors had clear borders with the adjacent sound enamel. Maxillary incisors and canines showed linear groves with a reduced localized thickness of the enamel and a smooth border with the normal enamel (Figure 1). The patient also reported that the first permanent mandibular molars on both sides often triggered sensitivity especially while eating. The clinical pattern of hypomineralized defects and relevant past medical history led to the diagnosis of MIH. The long data set form formulated by Ghanim et al. was followed to assess the clinical status and extent of MIH and other enamel defects (Tables 1 and 2).

### 2.1. Management

Three consecutive treatment stages were defined.

1. Preventive stage

- Appropriate dietary advice to avoid dental erosion and caries
- Professional topical application of arginine-containing desensitizing paste
- At-home oral hygiene practices using arginine-containing desensitizing toothpaste and mouthwash

(2) Aesthetic enhancement stage for anterior teeth

- Microabrasion
- Composite veneers

(3) Restorative stage for posterior teeth

- Full-coverage restoration using preformed stainless steel crowns (SSCs) for defective molars

**Figure 2:**
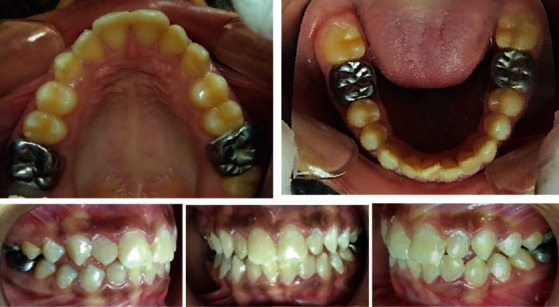
Postoperative intraoral photographs.

The preventive phase started by approaching the child and his parents with appropriate dietary advice. In-office topical application of desensitizing paste containing 8% arginine and calcium carbonate (Elmex sensitive professional desensitizing paste) was carried over for the affected mandibular first molars following the manufacturer's instruction. Simultaneously, the patient was instructed to use arginine-containing desensitizing toothpaste (Elmex sensitive professional toothpaste) and mouthwash (Elmex sensitive professional mouthwash) as a routine at-home oral hygiene practice. Aesthetic enhancement of anterior teeth was accomplished by opting for microabrasion and direct composite restorations for maxillary incisors, lower central incisors, and maxillary and mandibular canines. Complete composite veneering was done for more destructed lower lateral incisors. MIH-affected FPMs were restored with preformed SSCs as a full-coverage restorative approach (Figure 2).

## 3. Discussion

Amelogenesis is the process of dental enamel formation that can be separated into the initial phase of matrix formation through the secretion of matrix protein and later stages of mineralization and maturation. If any developmental disturbance occurs during the secretory phase, it results in a quantitative defect called hypoplasia. If a disturbance occurs in later stages of mineralization or maturation, it results in a qualitative defect called hypomineralization. MIH is a term that describes a qualitative defect of enamel presenting clinically as variable opacities concomitant with the distinct demarcation between the affected and sound enamel [2, 22].

The literature shows several possible etiological determinants which have been associated with MIH. They are generally identified as systemic factors during the pre-, peri-, and postnatal period, and they include genetic variations [23, 24, 25]. In the period from the final trimester of pregnancy to the third year of the child lies the phase of enamel maturation. Any disturbance to the enamel in this phase can lead to MIH [20, 26]. A considerable association has been found between MIH and premature birth, gastrointestinal system disorders, renal failure, rubella, various drug usage, and chickenpox [26, 27, 28, 29].. In this present case, the patient had a premature birth, was rhinovirus positive, and had CIPO with underlying neurological disease due to somatic mitochondrial disorder.

A minimally interventive conservative approach with microabrasion and the direct composite veneer was opted for all anterior teeth except mandibular lateral incisors similar to the treatment carried out by Pereira [30]. As the conservative approach is not meant to address the depth of destruction in the affected lower lateral incisors and salvage them, therefore, direct complete composite veneering was done for the lower lateral incisors.

Arginine paste is used as a dentinal tubule sealant and is recommended as desensitizing toothpaste in MIH-affected teeth. It has also been shown to block hydrodynamic pain mechanisms by reducing the number of exposed sensory afferent nerve endings [21, 31–33]. Hence, we used arginine-based paste in this case to treat MIH-related dentinal hypersensitivity.

In the present case, we have considered all four FPMs for restorative therapy. Based on the severity of damage due to MIH, there was a greater need for full-coverage restoration to restore the form, function, and longevity of the affected teeth. SSCs have been suggested considering their advantages as a treatment modality for severe MIH-affected molars [34]. SSCs can provide full coverage thereby maintaining the structural integrity of mutilated molars, prevents recurrent dental caries, improves oral hygiene, reduces dentinal hypersensitivity, restores crown height and occlusal functionality, and imparts longevity and high success [35–37]. This restorative approach of using SSCs for MIH-affected molars with extensive defects has been adopted in a few previous studies [38, 39]. However, long-term studies are needed to investigate the efficacy of SSCs. This can also be an interim restorative approach for MIH-affected molars in patients with the need of future orthodontic correction and extractions, to manage their malocclusion [38].

Weerheijm and colleagues defined MIH in 2001. They also raised speculation about the manifestation of MIH-like defects in the tips of permanent cuspids along with FPMs and PIs because they are mineralized in the same time frame as the FPMs and PIs. Following which studies were carried out to assess the extent of involvement of teeth other than FPMs and PIs, in a study, 27% of MIH-affected children who were in the permanent dentition stage showed one or more affected canines and premolars [3]. Another study recorded 25.7% MIH-affected individuals of age 14 years which show MIH-like defects in canines [11]. In a study on adolescents, 22.8% MIH-affected individuals of age 16 years reported having MIH-like defects in canine. They also recorded that maxillary canines were 2.3 times more frequently affected than mandibular canines [5]. In the present case, the defects were observed bilaterally in mandibular canines. Other enamel defects like hypoplasia, diffuse opacities, and amelogenesis imperfecta that are clinically similar to MIH defects can also occur due to any disturbances during amelogenesis. These abnormalities should not be misdiagnosed; instead, they are suggested to be assigned as present or absent allowing their distinction from MIH defects [15]. It has been suggested that systemic etiological factors acting for longer periods during enamel mineralization and maturation tend to produce more affected teeth with more severe defects. In the present case, MIH along with hypoplastic defects in maxillary anterior teeth was recorded following the long set data form for use in epidemiological studies on enamel hypomineralization [15].

MIH may lead to posteruptive enamel breakdown leading to dentine exposure, tooth sensitivity with increased caries susceptibility, behavioral management problems due to dental fear and anxiety, aesthetic complications, with a concomitant negative impact on child's self-esteem, personal relationship, and school performance, and an additional financial burden [13, 30, 40, 23]. Hence, early detection and comprehensive treatment of patients with MIH remain a priority.

## 4. Conclusion

This present case report to the best of our knowledge is the first case reported which acknowledges MIH in a patient with CIPO with underlying neurological disease due to somatic mitochondrial disorder. It also elicits the availability of various contemporary treatment options and their proper selection and early intervention to manage the functional and aesthetic problems caused by enamel defects and to improve the quality of life in the patients.

## Figures and Tables

**Table 1:** Assessment of the clinical status and extent of MIH along with other enamel defects in maxillary teeth.

Surface	Maxillary right							Maxillary left						
	17	16	15	14	13	12	11	21	22	23	24	25	26	27
Buccal/labial	A	0	0	0	12, I	12, I	12, I	12, I	12, I	12, I	0	0	0	A
Occlusal/incisal	A	3, II	0	0	0	0	0	0	0	0	0	0	3, II	A
Palatal	A	3, II	0	0	0	0	0	0	0	0	0	0	3, II	A

**Table 2:** Assessment of the clinical status and extent of MIH in mandibular teeth.

Surface	Mandibular right							Mandibular left						
	47	46	45	44	43	42	41	31	32	33	34	35	36	37
Buccal/labial	0	3, II	0	0	14, I	22, II	21, I	22, II	22, II	14, I	0	0	3, II	0
Occlusal/incisal	0	3, II	0	0	0	0	0	0	0	0	0	0	3, II	0
Lingual	0	3, II	0	0	0	0	0	0	0	0	0	0	3, II	0

## Data Availability

No data were used to support this study.
